# A questionnaire-based investigation of the swimming puppy syndrome: 115 dogs

**DOI:** 10.3389/fvets.2023.1233277

**Published:** 2023-08-21

**Authors:** Lea Rumpel, Petra Kölle, Monika A. Mille, Susanne K. Lauer, Yury Zablotski, Andrea Fischer

**Affiliations:** Centre for Clinical Veterinary Medicine, Ludwig-Maximilians-Universität München, Munich, Germany

**Keywords:** swimmer puppy, flat puppy, litter, growth, nutrition, weakness, paresis, breed predisposition

## Abstract

Swimming Puppy Syndrome (SPS) is a benign reversible condition of unknown etiology in multiple dog breeds. Affected dogs show laterally abducted limbs and are unable to stand and walk on their own. The current knowledge of this condition derives from few case reports or small case series. Therefore, the aim of this study was to collect data on the clinical course from a large cohort of dogs with SPS with an online questionnaire supported by video footage. Potential risk factors were compared between 110 litters with SPS and 103 unaffected litters. SPS was reported in 115 dogs from 48 different breeds comprising a wide range of small, middle, and large breeds. Litters with SPS were significantly smaller than unaffected litters. Cesarean sections were reported more frequently in affected litters, but the overall rate of reported birth complications did not differ significantly from unaffected litters. Most puppies were able to stand and walk at a median age of 4.5 weeks (up to 12 weeks) and clinical signs resolved at a median age of six weeks (up to 12 weeks). Puppies from large breeds showed faster recovery than puppies from medium and small breeds. Occasionally, residual deficits were reported and only three dogs failed to recover. A clustering of SPS occurred in closely related litters in four kennels of four different dog breeds (Greater Swiss Mountain Dog, Golden Retriever, Miniature Bull Terrier, Norwich Terrier). The study shows the benign clinical course of SPS in a large cohort of puppies from multiple dog breeds. Potential risk factors including reports on birth complications, size and muscle mass compared to littermates and diet of the dam during pregnany were evaluated and no influence on the occurrence of SPS was identified.

## 1. Introduction

The Swimming Puppy Syndrome (SPS) or Swimmer Syndrome has been described repeatedly in dogs in the veterinary literature in case reports or small case series (1–3). The largest report to this point includes 52 dogs with SPS in Thailand (2). The disorder received its name from the characteristic swimming movements of the affected limbs, and is also known as Flat Puppy Syndrome (4). Brachycephalic dog breeds, particularly English Bulldogs, are reported as being predisposed (2, 5). Male and female dogs are equally affected (1, 2). Similar conditions are reported in other animal species, e. g. cats, birds (spraddle leg syndrome), piglets and rabbits (splay leg syndrome) (6–9).

The signs of SPS are usually noticed by the breeders when puppies begin to walk at two to three weeks of age (2, 10). Puppies with SPS are unable to adduct the affected limbs, lie in sternal recumbency with laterally splayed limbs and show paddling movements when trying to move (3, 11). Neurological examination, complete blood count and serum chemistry are unremarkable except for a mild (two-fold) increase in creatine kinase compared to healthy puppies (3, 12, 13). Therefore, the diagnosis of SPS is usually based on the characteristic clinical signs (inability to stand and walk, abduction of affected limbs, paddling movements of the limbs resembling swimming movements when attempting to move) noticed in the first weeks of life and exclusion of other orthopedic (angular limb deformity, congenital elbow luxation, fracture, malunion) or neurologic (inflammation, brain or spinal cord malformation, trauma, early onset neurodegeneration) diseases *via* thorough clinical examination supported by further diagnostic methods (e. g. radiographs) (14). Physical therapy is frequently employed to help recovery, e. g. supported standing, passive movement and massages of the affected limbs, external support to prevent the legs from splaying as well as hobbling of the limbs (3, 5).

Information on the etiopathogenesis of SPS is scarce. Environmental, orthopedic and neurological factors have been implicated as causes for SPS (3, 5, 15). Furthermore, various hypotheses proposed that rapid weight gain, high bodyweight compared to littermates, nutritional or genetic factors could play a role in the development of the syndrome (1, 16–18). In piglets with a similar splay leg syndrome, a recent genome-wide association study identified four promising candidate genes but no causal gene variant were identified so far (19, 20). Similar attempts in dogs have failed (1, 21), and although there are first insights and clues to SPS in dogs, the underlying cause of this syndrome remains unknown.

Therefore, the aim of this present study was to collect data on affected breeds, clinical course, and risk factors for SPS by means of an internationally distributed questionnaire.

## 2. Materials and methods

A call for dogs with signs of SPS was published on the university website and distributed *via* social media and breeding clubs in German and English language to dog owners and breeders in Germany and other German and English-speaking countries in 2021. Respondents were asked to fill out an online questionnaire and to provide video footage of the affected puppies. A control group (unaffected litters) was recruited with a similar approach to compare potential risk factors (litter size, rate of birth complications, nutrition of the dam). Only litters with puppies without problems to stand or walk were included in this group. The study was conducted with ethical permission (AZ 207–27-3-2020).

### 2.1. Questionnaire

The questionnaire for puppies with SPS consisted of 96 questions and was structured in six categories. The categories included: 1: general information about the puppy (e. g. birth date, breed), 2: signs, 3: clinical course, 4: background information about the litter, 5: size of the puppy, 6: diet of the dam.

The questionnaire for the control group concentrated on questions about litter size, complications during pregnancy and birth as well as diet of the dam during pregnancy. The questions were reviewed by German and English native speakers (eight veterinarians and six dog owners or breeders) regarding understanding, phrasing, and structure of the questionnaire.

### 2.2. Inclusion and exclusion criteria

#### 2.2.1. Dogs with signs of SPS

Only dogs in which the owner stated that the dog was a Swimmer Puppy, and that the dog could not stand and walk on its own, showed the characteristic swimming movements with the affected legs and the characteristic onset of the signs of SPS were included in the study. Puppies which did not show swimming movements, or with an onset later than 28 days of age, or puppies, which were able to stand and walk before the first signs of SPS appeared, could not participate. Video footage provided further support of SPS when available. Videos were reviewed by two of the authors (LR, AF) who decided by consensus whether the dogs showed the characteristic signs of SPS and which limbs were affected.

#### 2.2.2. Control group (unaffected litters)

Only litters without any puppies with problems standing or walking could participate. The control group consisted of a large variety of breeds matching the distribution of breed sizes of the SPS group.

### 2.3. Diet of the dam during pregnancy

Information on feeding of the dam during pregnancy was collected with the questionnaire. The commercial brands which made a major contribution to the diet of the dams were analyzed using the software Diet Check Munich® (RV Software, Unterschleißheim, Germany). The requirements for adequate nutritional supply for pregnancy (regarding protein, carbohydrates, vitamins and minerals) were used as defined by the National Research Council (22).

### 2.4. Statistical analysis

The raw data of the questionnaires were descriptively analyzed in Microsoft Excel 2019 (Microsoft Corporation, Redmond, WA, United States). Statistical analyses were performed in R version 4.0.3 (The R Foundation for Statistical Computing, Vienna, Austria). Data were checked for normality with Shapiro–Wilk Normality Test. Not-normally distributed data were analyzed with nonparametric methods, such as Kruskal-Wallis and Mann–Whitney U test. Variances among groups were compared for homogeneity of variance with Levine’s test. Fisher’s Exact Test for Count Data was used to examine the relationship between affected front legs and the occurrence of a flat chest and to compare birth complications between healthy and affected litters. The association between selected factors (small vs. middle vs. large breed, all limbs vs. hind or front limbs only, the size and muscle mass of the affected puppy compared to its non-affected littermates), and the recovery time (weeks) were assessed using the Kruskal Wallis test. The breeds were categorized according to their bodyweight in large (> 25 kg), medium (10–25 kg) and small breeds (<10 kg). The student’s t-test was used to compare recovery times (weeks) between swimmer puppies of dams fed with or without nutritional deficient food. The Mann–Whitney U test was used to compare the recovery times between puppies with and without deformations, the litter size between litters with puppies with signs of SPS and unaffected litters from the control group, and the number of affected littermates between litters from dams with adequate nutrition vs. without adequate nutrition. Results were considered significant when the value of *p* was <0.05.

## 3. Results

### 3.1. Dogs with signs of SPS

134 questionnaires and video footage from 26 dogs were submitted. Nineteen questionnaires were subsequently excluded due to incomplete answers (*n* = 4), onset of clinical signs in older dogs (*n* = 4) and failure to describe swimming movements (*n* = 11). Thus, finally 115 questionnaires were available for evaluation. Review of videos confirmed the signs of SPS in all videos.

### 3.2. Control group (unaffected litters)

104 questionnaires of healthy litters without occurrence of SPS were submitted, whereof one was excluded because one puppy in this litter had problems walking. Thus, finally 103 questionnaires were available for evaluation.

### 3.3. Descriptive data

The 115 dogs with signs of SPS were born between 1972 and 2021 and originated from 110 litters (five siblings) and from 106 kennels. Forty-eight dog breeds were represented with 55 large breed dogs (47.8%), 36 medium breed dogs (31.3%) and 24 small breed dogs (20.9%). The most common dog breeds were Golden Retriever (14.8%; 17/115), French Bulldog (6.1%; 7/115), Labrador Retriever (4.3%; 5/115) and Pug (4.3%; 5/115). Six mixed-breed dogs were included. The questionnaires originated from 11 different countries, most of them from Germany (*n* = 49), United States (*n* = 26), Austria (*n* = 13) and the United Kingdom (*n* = 12) (Table 1).

**Table 1:** Comparison of potential risk factors between litters with swimmer puppies and unaffected litters.

	Litters with swimmer puppies	Unaffected litters	*p*
Number of litters	110	103	
Number of breeds	48	48	
Countries	Germany (*n* = 49) United States (*n* = 26) Austria (*n* = 13) United Kingdom (*n* = 12) Australia (*n* = 5) Canada (*n* = 4) Ireland (*n* = 1) Israel (*n* = 1) Norway (*n* = 1) South Africa (*n* = 1) Switzerland (*n* = 1) unknown (*n* = 1)	Germany (*n* = 64) United States (*n* = 11) Austria (*n* = 9) United Kingdom (*n* = 3) Switzerland (*n* = 3) Australia (*n* = 1) New Zealand (*n* = 1) unknown (*n* = 11)	
Breed size			
Large	47.3% (52/110)	55.3% (57/103)	
Medium	31.8% (35/110)	26.2% (27/103)	
Small	20.9% (23/110)	18.4% (19/103)	
Dog breeds	Golden Retriever (*n* = 16)	Golden Retriever (*n* = 16)	
	French Bulldog (*n* = 6) Mixed breed (*n* = 6)	Labrador Retriever (*n* = 6) German Shepherd (*n* = 6)	
	Labrador Retriever (*n* = 5) Pug (*n* = 5)	Bernese Mountain Dog (*n* = 5)	
	Chow-Chow (*n* = 4) Miniature Schnauzer (*n* = 4) Rottweiler (*n* = 4)	Chow-Chow (*n* = 4) Mixed breed (*n* = 4)	
	Cocker Spaniel (*n* = 3) Dachshund (*n* = 3) German Shepherd (*n* = 3) Greater Swiss Mountain Dog (*n* = 3) Staffordshire Bull Terrier (*n* = 3)	Newfoundland (*n* = 3) Shih Tzu (*n* = 3) Whippet (*n* = 3) Miniature Poodle (*n* = 3)	
	Bernese Mountain Dog (*n* = 2) Border Collie (*n* = 2) Cavalier King Charles Spaniel (*n* = 2) Goldendoodle (*n* = 2) Great Dane (*n* = 2) Miniature Bull Terrier (*n* = 2) Shetland Sheepdog (*n* = 2) Siberian Husky (*n* = 2) Westphalian Dachsbracke (*n* = 2)	Briard (*n* = 2) Border Collie (*n* = 2) Cocker Spaniel (*n* = 2) Dachshund (*n* = 2) Entlebucher Mountain Dog (*n* = 2) French Bulldog (*n* = 2) German Short Hair (*n* = 2) German Wire Hair (*n* = 2) Havanese (*n* = 2) Hovawart (*n* = 2) Lagotto Romagnolo (*n* = 2)	
	American Akita (*n* = 1) American Bully (*n* = 1) American Staffordshire Terrier (*n* = 1) Beagle (*n* = 1) Bichon Frise (*n* = 1) Bordeaux dog (*n* = 1) Boxer (*n* = 1) Cao da Serra da Estrela (*n* = 1) Continental Bulldog (*n* = 1) Dalmatian (*n* = 1) English Bulldog (*n* = 1) German Spaniel (*n* = 1) Havanese (*n* = 1) Irish Wolfhound (*n* = 1) Kerry Blue Terrier (*n* = 1) Labradoodle (*n* = 1) Lagotto Romagnolo (*n* = 1) Landseer (*n* = 1) Newfoundland (*n* = 1) Norfolk Terrier (*n* = 1) Norwich Terrier (*n* = 1) Old English Bulldog (*n* = 1) Pit Bull Terrier (*n* = 1) Pyrenean Mountain Dog (*n* = 1) Small Münsterländer (*n* = 1) Welsh Corgi Pembroke (*n* = 1) Yorkshire Terrier (*n* = 1)	American Bulldog (*n* = 1) Australian Cattle Dog (*n* = 1) Australian Shepherd (*n* = 1) Basenji (*n* = 1) Beagle (*n* = 1) Berger des Pyrenees (*n* = 1) Bobtail (*n* = 1) Cavalier King Charles Spaniel (*n* = 1) Chihuahua (*n* = 1) Collie (*n* = 1) Dalmatian (*n* = 1) Doberman (*n* = 1) English Springer Spaniel (*n* = 1) German Spaniel (*n* = 1) Kuvasz (*n* = 1) Markiesje (*n* = 1) Miniature Dachshund (*n* = 1) Miniature Schnauzer (*n* = 1) Nederlandse Kooikerhondje (*n* = 1) Papillon (*n* = 1) Pomeranian (*n* = 1) Rhodesian Ridgeback (*n* = 1) Rottweiler (*n* = 1) Samojede (*n* = 1) Shetland Sheepdog (*n* = 1) Soft Coated Wheaten Terrier (*n* = 1) Spanish Water Dog (*n* = 1) Wirehaired Slovakian Pointer (*n* = 1)	
Mean litter size	4.2 puppies/litter	6.4 puppies/litter	< 0.001
Birth complications	38.2% (42/110)	35.9% (37/103)	0.748
cesareans	13.6% (15/110)	4.9% (5/103)	0.034
still births	22.7% (25/110)	29.1% (30/103)	0.211

In the control group (unaffected litters), 103 litters were analyzed from 48 different breeds. Four mixed-breed dogs were included. The control litters were born between 2000 and 2022. Fifty-seven large breed dogs (55.3%), 27 medium breed dogs (26.2%) and 19 small breed dogs (18.4%) were included. The most common breeds were Golden Retriever (15.5%; 16/103), Labrador Retriever (5.8%; 6/103), German Shepherd (5.8%; 6/103) and Bernese Mountain Dog (4.9%; 5/103). The litters from the control group originated from seven different countries, mostly from Germany (*n* = 64), United States (*n* = 11) and Austria (*n* = 9) (Table 1).

### 3.4. Signs of SPS

**Figure 1:**
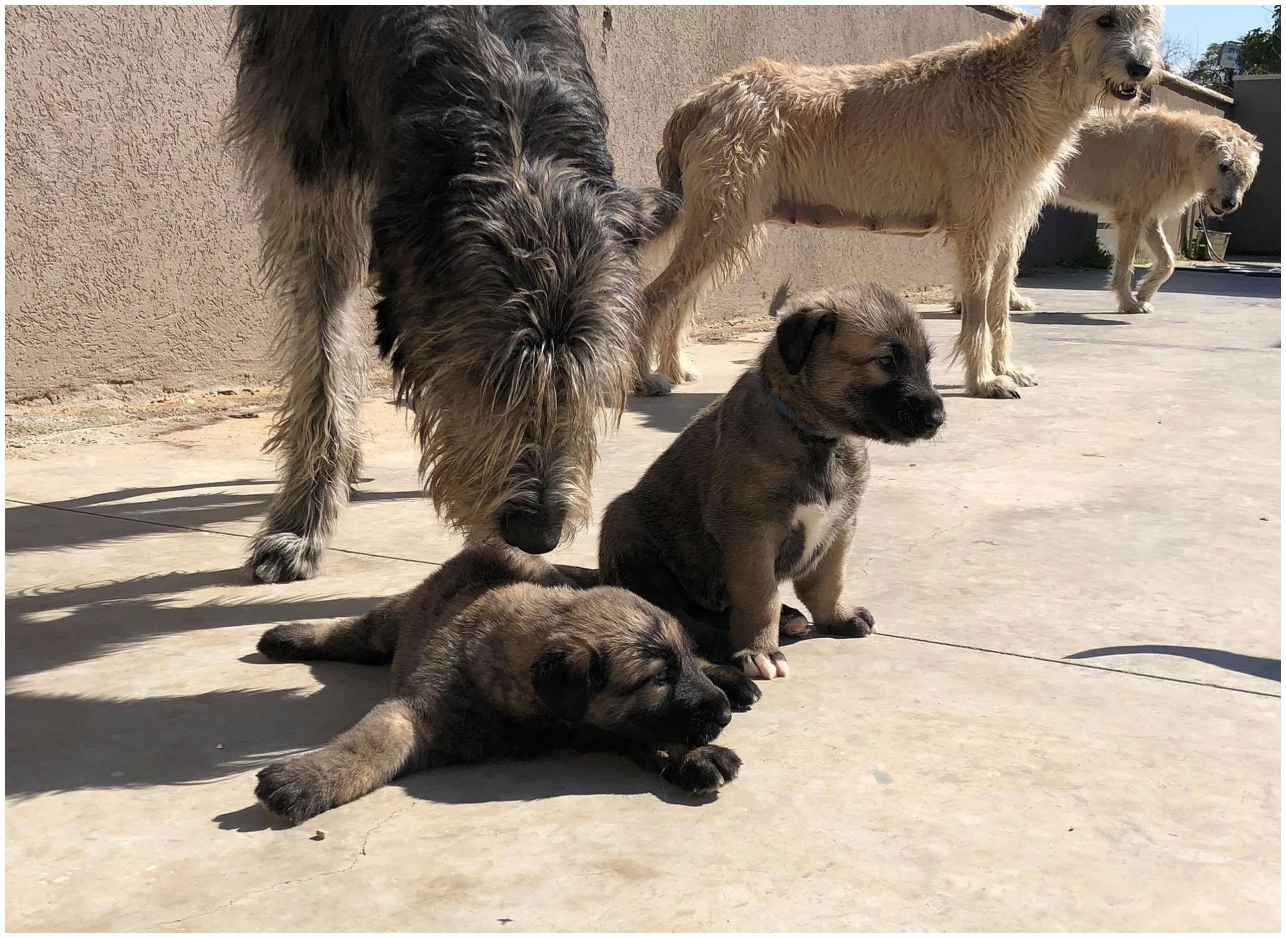
Swimmer puppy from a large breed (Irish Wolfhound, male, 3 weeks). The puppy was able to stand and walk at 4 weeks of age (printed with permission of the dog owner).

The dogs showed the first signs of SPS at a median age of 14 days (mean 13.6, range 1–28). All limbs were affected in 56 dogs (48.7%) (as shown in Figure 1), only the hind limbs in 41 dogs (35.7%) and only the forelimbs in 13 dogs (11.3%). In five puppies limbs were asymmetrically affected (no videos available). The owners reported deformations in 61 of the swimmer puppies (53.0%) with a flattened thorax (58 dogs) and leg deformations (eight dogs) reported most frequently (five dogs had both). A flattened thorax was significantly more often reported in swimmer puppies with affected forelimbs (all limbs or fore limbs only) compared to swimmer puppies with only affected hindlimbs (*p* = 0.005).

The owners considered 49 swimmer puppies (43.4%; 49/113) bigger than their littermates (18 slightly bigger and 31 markedly bigger), 41 swimmer puppies (36.3%; 41/113) had the same size as their littermates and 23 swimmer puppies (20.4%; 23/113) were smaller than their littermates (14 puppies slightly smaller and nine markedly smaller). This information was unavailable for two dogs. Owners described the muscle mass of swimmer puppies as more prominent in four puppies (3.8%; 4/105) and less prominent compared to littermates in 53 puppies (50.5%; 53/105). No difference was noticed in 48 puppies (45.7%; 48/105), and information was missing for ten dogs.

Physiotherapy was performed by the owner, physiotherapist, or a veterinarian in 107 swimmer puppies (93.0%). Commonly applied methods were taping of the splayed legs, placing the puppies on their sides during sleep and rest, change to a non-slippery or uneven ground and passive movement of the limbs. Most owners (97.1%; 101/104) had the impression that physiotherapy led to an improvement of the signs of SPS. Three owners failed to answer this question.

### 3.5. Clinical course

The swimmer puppies were able to stand and walk on their own at a median age of 4.5 weeks (range 2–12, mean 4.9). At the median age of six weeks (range 2–12, mean 6) the puppies were free or almost free of signs of SPS. When considering only dogs older than 12 weeks at the time of data acquisition, then 97.0% (97/100) had completely or largely recovered.

Overall, 109 dogs with SPS (94.8%; 109/115) had recovered at the time of data acquisition. The signs of SPS had disappeared completely (86.2%; 94/109) or almost completely with only mild residual deficits reported in 15 dogs (13.8%; 15/109). Residual deficits were intermittent slipping on smooth or wet surface when walking, a slightly unsteady gait, a wider stance, and a minor paw deformation. Clinical signs were still present at the time of data collection in six dogs (5.2%; 6/115; American Bully, American Bulldog, French Bulldog, Goldendoodle, Pug, Landseer) at a median age of 10.5 weeks (range 23 days – 4.5 years). Three dogs were younger than 12 weeks (three, six and seven weeks old) and therefore in the age range where recovery is still likely, while three other dogs (2.6%; 3/115) were considered as failed to recover: The Landseer was euthanized at seven months of age because it was still unable to stand and walk without assistance. His owner reported on poorly developed acetabula on radiographs. The pug (4.5 years old) was able to stand and walk on its own but had deformed elbow joints. The American Bully (14 weeks old) was only able to crawl and unable to stand and one tarsal joint was reported to be dislocated.

**Figure 2:**
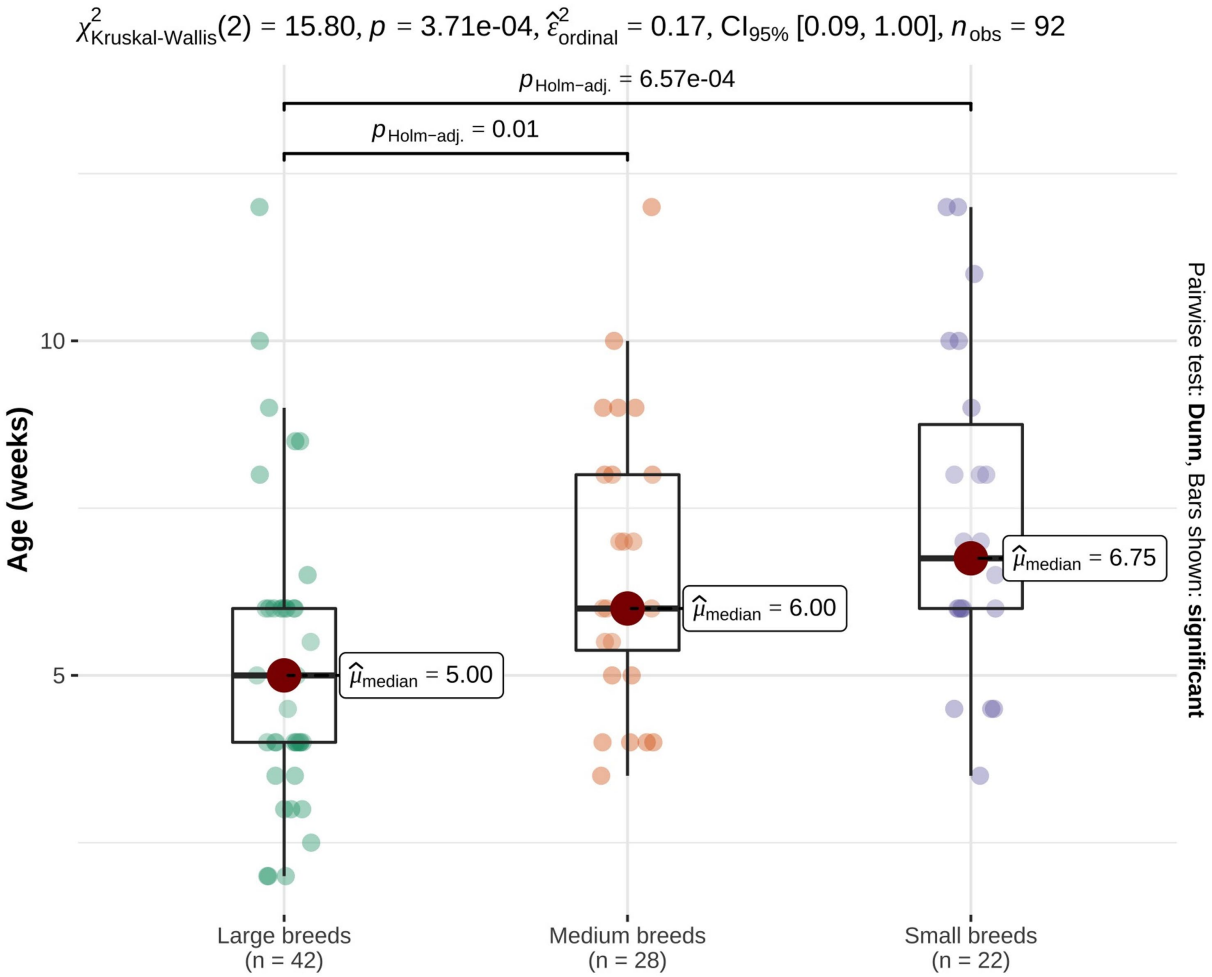
Recovery time of swimmer puppies. Swimmer puppies from large breeds recovered significantly faster than puppies from medium or small breeds.

Factors potentially affecting recovery time were evaluated for their influence on shortening or extending the time until recovery. Large breed swimmer puppies recovered significantly faster compared to medium breed (*p* = 0.010) and small breed swimmer puppies (*p* < 0.001, Figure 2). Recovery time was not affected by the following factors: number of affected limbs (all limbs; only fore limbs or only hind limbs, *p* = 0.880), presence of deformations (*p* = 0.440), physiotherapy (*p* = 0.600), difference in size (*p* = 0.110) or muscle mass compared to littermates (*p* = 0.410).

### 3.6. Affected litters

**Figure 3:**
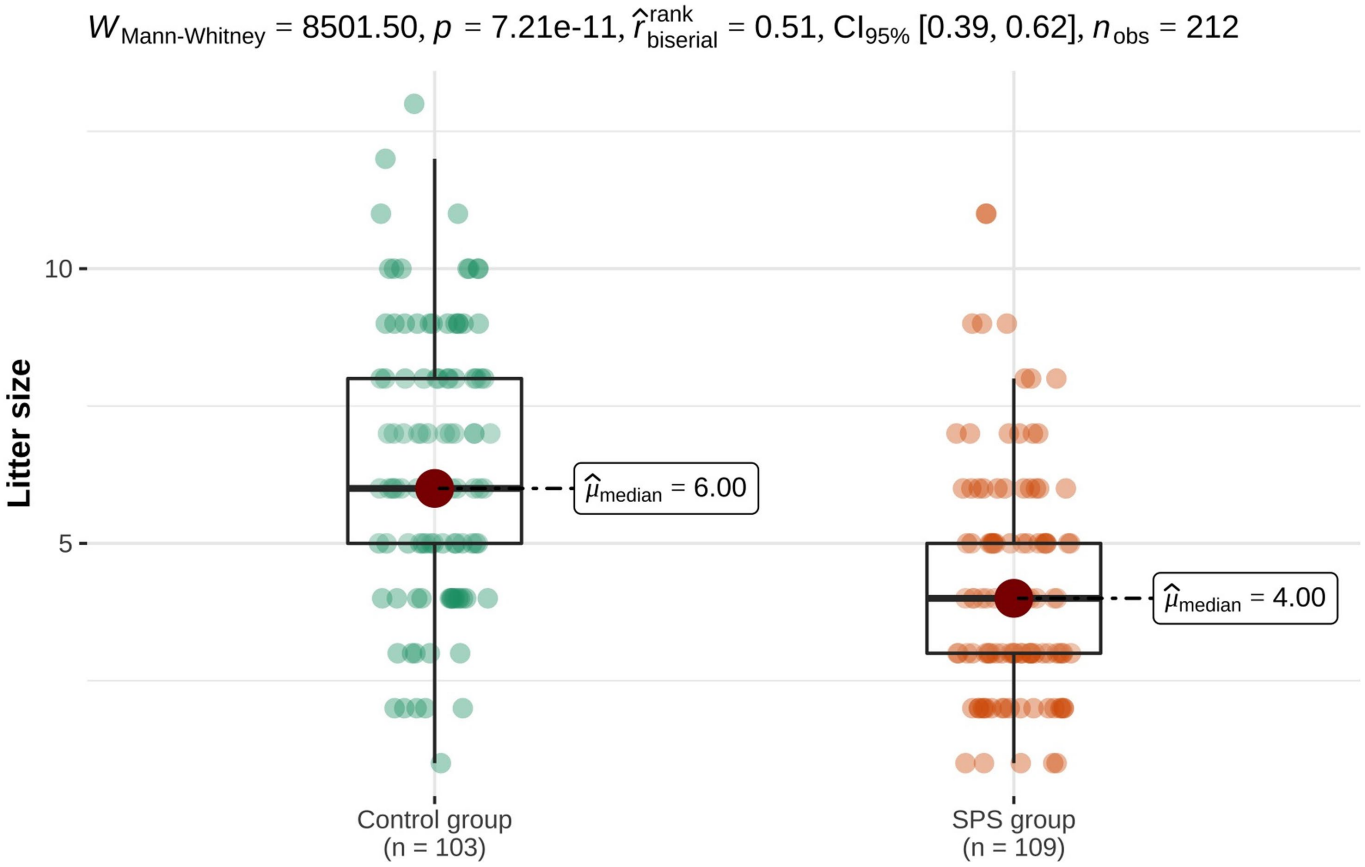
Comparison of litter size between litters with swimmer puppies and unaffected litters. Litters with swimmer puppies were significantly smaller than unaffected litters.

The mean number of puppies with SPS in each litter was 1.4 (range 1–5, median 1). In 76 litters (71.0%) only one single puppy showed signs of SPS (76/107), while in 31 litters (29.0%) two or more puppies were affected (31/107). In ten litters, the entire litter was affected with SPS and each dog within the litter showed signs of SPS (10/31). No information on affected littermates was available for three litters. Five litters in the SPS group (4.5%) consisted only of one puppy (5/110). For litters with swimmer puppies, the median litter size was four (range 1–11, mean 4.1). Significantly larger litters were reported in the control group (median six dogs per litter; range 1–13, mean 6.4; *p* < 0.001; Figure 3).

The age of the dams at parturition was known for 109 litters with SPS. The median age was three years (range 2–7, mean 3.4). In the control group, the median age of the dams was four years at parturition (range 1–8, mean 3.6). The Body Condition Score (BCS) of the dam before pregnancy was evaluated for 95 dams by the owners using a scale from one to nine. The median BCS of dams with SPS litters was five (range 1–9, mean 4.7). Birth complications were reported in 42 litters with SPS (38.5%; 42/109), while in 67 SPS litters (61.5%) no complications had occurred (67/109). Information on birth complications was missing for one litter. Complications were mostly stillbirths (25 litters) or cesarean sections (15 litters). In the control group, birth complications were reported in 37 litters (35.9%), mostly stillbirths (30 litters) or cesarean sections (5 litters). There was no difference in the rate of birth complications between affected and unaffected litters (*p* = 0.776), but significantly more cesarean sections were reported in litters with swimmer puppies (*p* = 0.034; Table 1).

### 3.7. Diet of the dam during pregnancy

Information about the feeding of the dam during pregnancy was provided for 101 affected litters. Ninety-six dams (95.0%; 96/101) were mainly fed with commercial food brands. Of these, 28 dams (29.2%; 28/96) were additionally fed a raw meat or a home-cooked diet. Five dams (5.0%; 5/101) were exclusively fed a raw meat diet. In the control group (unaffected litters), slightly less dams (81.6%, 84/103) were mainly fed with commercial food brands (*p* < 0.004). Of these, 18 dams (21.4%; 18/84) additionally received raw meat or a home-cooked diet. Nineteen dams (18.4%; 19/103) were exclusively fed a raw meat or self-cooked diet.

The brands of the commercial food were known for 80 dams in the SPS group. Most dams were fed with well-known brands. Reviewing the food brands *via* Diet Check Munich© according to the information provided by the food companies for each brand revealed that the food brands of 61 dams (76.3%; 61/80) were meeting the requirements for pregnancy according to the National Research Council. The food brands of 19 dams (23.8%; 19/80) showed nutritional deficiencies in the declared ingredients. Minor deficiencies in iron and iodine as well as copper and zinc were most common. Protein amount was sufficient for pregnancy in all diets. There was no difference in the number of swimmer puppies between litters from dams fed with commercial food brands with or without minor nutritional deficiencies (*p* = 0.980). There was also no difference in recovery time (*p* = 0.300).

### 3.8. Information on related litters

Information on related litters was available for 94 swimmer puppies. The owners of eight swimmer puppies (8.5%; 8/94) reported on swimmer puppies in related litters (siblings excluded). The other owners (91.5%; 86/94) were not aware of any related dogs with similar signs. The eight puppies with known affected relatives in other litters originated from seven litters and from four different kennels. Each of the eight puppies had fully recovered from SPS. One Greater Swiss Mountain Dog breeder (four dogs from that kennel participated in this study) reported on one dam with three litters with swimmer puppies. The half-sister of this dam (same mother, different father) also had one litter with swimmer puppies. Both dams also had healthy litters without occurrence of SPS. In another breeding of Golden Retrievers (one dog in this study), the breeder indicated one dam as the source of SPS. The dam had four litters with swimmer puppies and two of her daughters (who did not have SPS themselves) also had litters with swimmer puppies. One breeder of Norwich Terriers (one dog in this study) reported on the occurrence of SPS in litters of different dams but provided no information about the sires. In another breeding of Miniature Bullterriers (two dogs in this study), a sire had two litters with swimmer puppies with different dams.

## 4. Discussion

The data from 115 dogs with signs of Swimming Puppy Syndrome in this questionnaire study indicate that SPS occurs in a wide variety of breeds from many geographic regions. Furthermore, our results confirm an overall good prognosis.

Previous studies reported brachycephalic breeds to be predisposed to SPS (2, 5). Similarly, in our study, two brachycephalic breeds (French bulldogs and pugs) were among the four most common breeds with SPS. The frequent description of SPS in Golden Retrievers and Labrador Retrievers in our cohort is probably related to the popularity of these breeds. The Golden Retriever was also the most common breed in the control group. Other authors had similar observations and reported the frequent occurrence of SPS in Golden Retrievers and Labrador Retrievers (1, 2). However, other breeds appeared more affected (1, 2).

Our data support an association between SPS and litter size. SPS-affected litters were smaller than the litters of the control group, thus confirming observations in earlier studies (1, 2). Frequently only one puppy per litter was affected, but in some litters more than one puppy showed clinical signs of SPS and on some occasions all puppies within a litter were affected by SPS. More cesarean sections were reported in litters with SPS compared to control litters, but the overall rate of birth complications was similar in both groups. The higher rate of cesarean sections in litters with SPS may be due to the smaller litter size in this group, and thus a higher risk for dystocia (23). Larger puppies are also more likely to cause problems during birth (24). Moreover, brachycephalic breeds, that were overrepresented in the SPS group, are also more likely to have cesarean sections (23, 25). These factors might contribute to the higher number of cesarean sections in the SPS group.

Previous observations from other authors indicated that larger and heavier puppies and puppies with an excessive weight gain were more frequently affected by SPS (1, 2). Accordingly, many puppies in our study (43.4%) were reported to be larger than their siblings. On the other hand, some puppies (20.4%) were rated smaller than their siblings or showed no difference in size (36.3%). In line with these observations, other authors also described SPS in puppies of smaller size (12).

The clinical signs of SPS reported in the dogs from this cohort and previous reports are indicative of weakness, decreased muscle tone and strength resulting in increased limb abduction. Behavior and mentation were described as normal in the swimmer puppies from our cohort. Thus, peripheral weakness consistent with a disorder of the muscles, neuromuscular junction, peripheral nerves, or motor neurons needs to be taken into consideration. Approximately half of the puppies were reported to have less prominent muscle mass than their siblings. Almost half of the dogs (48.7%) showed swimming movements with all limbs, and 35.7% only with the hind limbs. These observations are in line with a Brazilian study, that also mainly reported puppies with clinical signs of SPS in all limbs (18). In contrast, earlier studies and case reports reported that the hindlimbs were predominantly affected (2, 3). A large number of puppies in our study showed a flattened thorax, which may be the consequence of immobility (11, 17). Floppy infant syndrome in human newborns shares clinical similarities with SPS. It is characterized by general hypotonia presenting as abnormal frog-like postures, reduced resistance to passive movement and excessive range of joint movement (26, 27). It can be caused by multiple disorders of the muscles, the peripheral and most commonly the central nervous system (28, 29). A self-limiting and remitting course is characteristic for benign congenital hypotonia.

SPS in dogs also shares similarities with the splay leg syndrome in piglets. Affected piglets are also unable to stand and walk shortly after birth, and the affected limbs are splayed laterally (6). Later development of hindlimbs during embryogenesis has been suggested as the cause for predominantly affected hindlimbs in splay legged pigs (6). Due to the greater economic impact, more research has been performed on porcine splay leg syndrome. Abnormal and delayed development of muscle fibers has been described as a potential cause for the syndrome as well as a myofibrillar hypoplasia, possibly associated with incompletely matured muscles (6, 30–32). There are only rare reports of postmortem histopathologic examinations of swimmer puppies in the veterinary literature due to the self-limiting character of the disease. So far, pathological findings have been inconclusive (4, 33). There are several hypotheses in the literature: Nganvongpanit et al. proposed, that SPS might represent a metabolic muscle disorder leading to muscle atrophy or hypoplasia, which would explain the less prominent musculature in puppies with SPS (2). Other authors discussed delayed and prolonged muscle development as cause for SPS (3, 5, 15). However, the inability of swimmer puppies to walk properly could also result in decreased muscle mass. Therefore, decreased muscle mass in swimmer puppies could also be a consequence of immobility, and not part of the original problem.

Interestingly, we observed an increased incidence of SPS in four kennels of four different breeds: Greater Swiss Mountain Dog, Golden Retriever, Norwich Terrier, and Miniature Bull Terrier. These included reports on dogs which appeared to produce swimmer puppies repeatedly in subsequent litters. Yet, breeding of these animals did not always result in litters with affected puppies and the number of affected puppies varied. Sometimes only some puppies of the litter showed signs of SPS, and sometimes all puppies within a litter were affected. Signs of SPS were reported in male as well as female dogs. In a previous study investigating the heritability of SPS in a population of Labrador Retrievers with 16 affected dogs, data from the analysis of the pedigrees supported a genetic influence on the occurrence of SPS (1). Another retrospective study from Brazil reported that 62% of 26 puppies with SPS were related to other affected puppies (18). Genetic investigation of four affected and three healthy Siberian Huskies failed to document any genetic variation (21). Recent investigations on the genetic background of splay leg syndrome in pigs identified potential candidate genes, but no causal gene variants (19, 20). In rabbits, splay leg syndrome presents with similar clinical signs and is considered a genetic disorder influenced by environmental factors (34). In a colony of rabbits, it was possible to completely eliminate the occurrence of splay leg syndrome by removing the affected animals and suspected genetic carriers (35). Our data support a genetic background of SPS in several kennels of different breeds. However, predisposing factors from the kennel’s environment could also play a role in the clustering.

Our questionnaire study asked for information on long-term outcome. It is notable that most dogs recovered completely with no or only minor residual deficits in a few dogs. These results confirm previous reports on the overall benign clinical course of SPS and characterize SPS as a reversible disorder. In our study, the puppies were able to stand and walk without support at the mean age of 4.5 weeks. By the age of three months (12 weeks), almost all puppies had largely or completely recovered. On rare occasions (three dogs), the owners reported that the dogs failed to recover despite initial consideration of SPS. Two of these dogs had deformations of the limbs, namely deformed elbow joints and a dislocated tarsal joint. It remains unclear whether these deformities developed as a consequence of severe SPS, immobility and abnormal posture of the limbs and joints over prolonged periods, or whether the deformations were already present at birth. Ramos et al. also reported three puppies with congenital subluxation of the elbow joints (18). These observations suggest that puppies with suspected SPS should be thoroughly assessed by a veterinarian for any deformities of the limbs which could interfere with recovery, and the need for special treatment or a preventative approach. These deformities could also mimic the signs of SPS which might lead to misdiagnosis of SPS.

Regarding recovery, the size of the breed was the only factor predicting the speed of recovery. Puppies from large breeds recovered significantly faster than puppies from medium and small sized breeds, with dogs from small breeds being the slowest to recover. Small and large breed dogs express general differences in growth and development. Puppies from large breeds display a more rapid growth as they need to achieve a larger form (36, 37). This is partly due to higher growth hormone concentrations stimulating the growth of the musculoskeletal system (38). This may help puppies with SPS from larger breeds to stand and walk earlier than puppies from smaller breeds. The affected legs (front legs, hind legs, or all legs) had thereby no influence on recovery time and neither did the presence of a flattened thorax. A less prominent muscle mass was also not associated with a longer recovery time, and the same applies to the size of the puppies compared to their siblings.

Most participants of this study reported that they had performed physiotherapy and were content with the outcome. Most commonly, the affected legs were taped together to avoid lateral abduction. This method has also often been described in previous cases in dogs as well as pigs as an effective therapy (3, 39). Other methods like placing the puppies on their sides while sleeping might help to prevent a secondary flattening of the thorax. Switching to a rougher surface with more grip helps the puppies to place their feet in a normal stance. Because no on-site clinical examinations were possible, the conclusions on the effect of physiotherapy in the study population were limited. Nevertheless, performing physical therapy and assisting with movements could prevent deformations and complications associated with the non-ambulatory state and the immobility of swimmer puppies. It also prevents muscle shortening and helps to maintain the mobility of the joints (40). Other authors promoted the benefits of an early start with physiotherapy (3). Therefore, we encourage breeders and owners to start physiotherapy with swimmer puppies as early as possible. Physiotherapy and exercise is also suggested for children with congenital hypotonia to prevent muscle shortening and improve motor function (41, 42).

Interestingly, most dams were fed with high-quality commercial food that met the requirements for pregnancy according to the NRC. The dams which received food not fully meeting the requirements did not produce litters with more affected puppies compared to dams fed according to NRC requirements. Other nutritional factors like excessive milk intake and a rapid weight gain are discussed by others as potential causes for SPS (1, 17). We did not evaluate the nursing and weight gain of the puppies, but our cohort also included swimmer puppies that were the same size or even smaller than their littermates. In pigs, choline deficiency in the sow’s diet is discussed as a potential underlying cause for the splay leg syndrome, but this has not yet been confirmed (6, 39). The choline content of the dams’ diets was not available in this study.

## 5. Limitations and future directions

A limitation of this study is the lack of on-site neurological and orthopedic examinations of the affected dogs and the limited number of available videos. Yet, review of the submitted videos confirmed the presence of clinical signs compatible with SPS in each case. Swimmer puppies were included based on the characteristic clinical signs, and we were not able to rule out other diseases which could present with a similar phenotype, because many puppies had not been presented to a veterinarian or the medical records were not available for review. Furthermore, breeders might be biased when answering certain questions (e. g. hereditary diseases) about their kennels.

The evaluation of the diet of the dams during pregnancy was based on optimal caloric intake and the data provided by the food companies for their brands. For more precise calculations, exact analysis of the diet’s nutrients, the actual amount of the food and the weight of the dam should be considered.

For further investigation of the etiology of SPS studies with electrodiagnostic analysis and muscle and nerve biopsies for investigation of peripheral causes of weakness (including evaluation of muscle fiber type composition, peripheral nerve, nerve roots and neuromuscular endplates), as well as investigations of muscle metabolism, gene expression profiles and investigations for reversible metabolic defects are possible tools.

## 6. Conclusion

Our data show that SPS affects puppies from many breeds. We confirmed that SPS is a benign reversible condition in the majority of dogs. Most puppies recovered before 12 weeks of age. Affected dogs usually come from smaller litters. Dogs from larger breeds recovered faster than dogs from smaller breeds. The reports suggest that the diet of the dam probably has no major influence on the incidence of SPS. A genetic predisposition was suggested in some litters. Therefore, further investigations with metabolic and genetic profiling of affected dogs and litters may be indicated. Furthermore, affected puppies should be examined thoroughly to recognize any limb deformities as cause or consequence of SPS.

## Data availability statement

Data sets are available on request. The raw data supporting the conclusions of this article will be made available by the authors, without undue reservation.

## Author contributions

The questionnaire was designed by LR and reviewed by AF, PK, MAM and SKL. LR performed the study. YZ and LR performed the statistical analysis. LR and PK performed the Diet Check. LR wrote the manuscript. AF, PK, MAM, SKL and YZ reviewed the manuscript. All authors contributed to the article and approved the submitted version.

## Conflict of interest

The authors declare that the research was conducted in the absence of any commercial or financial relationships that could be construed as a potential conflict of interest.

## Publisher’s note

All claims expressed in this article are solely those of the authors and do not necessarily represent those of their affiliated organizations, or those of the publisher, the editors and the reviewers. Any product that may be evaluated in this article, or claim that may be made by its manufacturer, is not guaranteed or endorsed by the publisher.
